# Impact of SGLT2 Inhibitors on Preventing Heart Failure Hospitalizations in Colombian Patients With Uncontrolled Type 2 Diabetes Mellitus

**DOI:** 10.7759/cureus.77725

**Published:** 2025-01-20

**Authors:** David Alexander Vernaza Trujillo, Lendy Yaneth Rojas Bautista, Claudia Marcela Ramirez Espinosa, Santiago Sierra Castillo, David Corredor-Rengifo, David Aristizabal Colorado, Alin Abreu Lomba

**Affiliations:** 1 Epidemiology, Fundación Universitaria del Área Andina, Bogotá, COL; 2 Interinstitutional Group of Internal Medicine 1 (GIMI1), Universidad Libre, Cali, COL; 3 Medicine, Universidad CES, Medellín, COL; 4 Internal Medicine, Universidad Libre, Cali, COL; 5 Endocrinology, Clínica Imbanaco, Cali, COL

**Keywords:** dapagliflozin, empagliflozin, glycemic control, heart failure hospitalization, hypertension, sglt2 inhibitor, type 2 diabetes mellitus (t2dm)

## Abstract

Objective: This study aims to evaluate the effects of SGLT2 inhibitors, specifically empagliflozin and dapagliflozin, on the prevention of heart failure hospitalizations and the improvement of metabolic control in patients with type 2 diabetes mellitus (T2DM) without documented high cardiovascular risk. The study aimed to assess the impact of these treatments on glycemic control, blood pressure, weight, and cardiovascular outcomes over an 18-month follow-up period in a Colombian population.

Materials and methods: A retrospective cohort study was conducted with 122 patients with uncontrolled T2DM at the Clínica Imbanaco in Cali, Colombia. Five treatment groups were identified, including various metformin combinations with other agents intensified with empagliflozin and dapagliflozin. Patients were retrospectively followed for 18 months, assessing treatment effects on the first hospitalization due to heart failure, glycemic control, blood pressure, and body weight. Multivariate repeated-measures ANOVA was used to analyze clinical variable changes over time. Additionally, Kaplan-Meier survival analysis estimated the cumulative probability of hospitalization for each treatment group, and Cox regression evaluated associations between different treatments and the risk of heart failure hospitalization.

Results: Patients treated with metformin + empagliflozin showed a significant reduction in HbA1c levels, from an initial mean of 7.75% to 6.77% at the end of follow-up (-0.97%; 95% CI: -1.31 to -0.63, p < 0.001) compared to baseline. Blood pressure in the empagliflozin group also showed significant decreases. Final systolic blood pressure reached an average of 120.40 mmHg (95% CI: -22.63 to 1.54, p > 0.05), reflecting a -10.55 mmHg reduction from baseline. Diastolic blood pressure decreased to an average of 78 mmHg (95% CI: -10.71 to -0.69, p < 0.05), with a reduction of -5.7 mmHg compared to baseline. Regarding hospitalizations, Cox regression analysis indicated an HR of 0 for the empagliflozin group (p < 0.001), with no reported heart failure hospitalizations during the study period.

Conclusions: Analysis of left ventricular ejection fraction and first heart failure hospitalization in patients with T2DM treated with SGLT2 inhibitors reveals that empagliflozin is not only effective in glycemic control, weight management, and blood pressure reduction but also shows preventive potential against heart failure progression, even in patients without high cardiovascular risk. These findings, aligned with evidence from classical studies, suggest that empagliflozin should be considered in the early management of T2DM to reduce heart failure incidence and improve long-term outcomes.

## Introduction

Type 2 diabetes mellitus (T2DM) is one of the most prevalent and rapidly growing chronic conditions globally, and Colombia is no exception to this trend. According to the 10th Edition of the International Diabetes Federation Atlas, approximately 537 million adults were living with diabetes in 2021, with projections estimating this number will rise to 643 million by 2030 and 783 million by 2045. This represents a significant public health challenge [1]. In Colombia, T2DM prevalence is estimated to range between 4% and 8%, with particularly high rates in urban areas among individuals aged 20 to 69 years, affecting approximately 3.4 million adults [2-6].

Cardiovascular complications are the leading cause of morbidity and mortality in T2DM patients. Evidence shows that cardiovascular risk begins to increase during the early stages of dysglycemia, such as prediabetes, predisposing patients to major cardiovascular events, including heart failure. Approximately 40% of patients present macrovascular complications at the time of T2DM diagnosis. Globally, studies such as EMPA-REG OUTCOME have reported a 35% reduction in heart failure hospitalization risk in high cardiovascular risk T2DM patients treated with empagliflozin compared to placebo. Similarly, the DAPA-HF and EMPEROR-reduced trials demonstrated significant reductions in hospitalization risk in patients with heart failure with reduced ejection fraction (HR = 0.75 and HR = 0.70, respectively) [6-21].

In Colombia, the Colombian Heart Failure Registry has shown that T2DM patients have a threefold higher risk of developing heart failure compared to non-diabetic individuals. The hospitalization rate for heart failure has also increased by 50% over the past five years [5]. Additionally, the EMPEROR-preserved and DELIVER studies documented 16-20% reductions in heart failure hospitalizations in patients with preserved ejection fraction treated with SGLT2 inhibitors, suggesting significant benefits even in patients without severe ventricular dysfunction [21-25].

Despite these findings, most studies on SGLT2 inhibitors have focused on populations with high cardiovascular risk. There is a lack of research on patients with uncontrolled T2DM and without advanced cardiovascular comorbidities who might benefit from preventive SGLT2 inhibitor interventions. This study aims to address this gap by evaluating the effects of empagliflozin and dapagliflozin in Colombian patients with T2DM without declared high cardiovascular risk, assessing their effectiveness in reducing hospitalizations and improving other key clinical indicators. Early identification and treatment of risk factors in this population are crucial to reducing morbidity and mortality and improving long-term outcomes [25].

## Materials and methods

This retrospective cohort study included 122 adult patients diagnosed with uncontrolled T2DM treated at Clínica Imbanaco in Cali, Colombia. Five treatment groups were identified, each receiving a different combination of metformin and other pharmacological agents, all intensified with an SGLT2 inhibitor. The groups were as follows: metformin + glibenclamide + dapagliflozin (n = 25), metformin + saxagliptin + dapagliflozin (n = 25), metformin + exenatide + dapagliflozin (n = 23), metformin + insulin + dapagliflozin (n = 29), and metformin + empagliflozin (n = 20).

Study design and procedures

Patients were followed for 18 months, with clinical evaluations conducted at four specific time points: baseline and at six, 12, and 18 months. Clinical variables included glycated hemoglobin (HbA1c), systolic blood pressure (SBP), diastolic blood pressure (DBP), and body weight. Each patient underwent a transthoracic echocardiogram during the study period, with left ventricular ejection fraction (LVEF) recorded as a crucial measure of ventricular function and its relationship to heart failure risk.

Inclusion and exclusion criteria

Patients were eligible if they met the following criteria: a diagnosis of uncontrolled T2DM (defined as HbA1c ≥7%), no history of heart failure, no severe cardiovascular comorbidities, and an echocardiogram with recorded ejection fraction during the study period. Patients in critical condition or with active malignancies were excluded to minimize potential confounding factors and maintain homogeneity in clinical characteristics within the cohort.

Hospitalization record

Hospitalizations due to heart failure were systematically recorded during the follow-up period. This event was defined as any hospital admission resulting from signs and symptoms of heart failure, confirmed by a medical professional and documented in the patient's medical history. This record was essential for survival analysis and risk estimation across treatment groups.

Statistical analysis

The statistical analysis was designed to evaluate changes in clinical variables over time and compare the effectiveness of the different treatment regimens. Multivariate repeated-measures ANOVA was used to assess differences in clinical measures (HbA1c, SBP, DBP, and weight) across time points and between groups, with Greenhouse-Geisser corrections applied when violations of sphericity were detected. This approach identified significant variations in glycemic control, blood pressure, and body weight among treatment groups.

For survival analysis of time to the first hospitalization due to heart failure, the Kaplan-Meier method was used to estimate cumulative hospitalization probability for each treatment group. Kaplan-Meier curves were compared using the log-rank (Mantel-Cox) test to assess differences in hospitalization-free survival among groups.

A Cox regression model was also employed to evaluate associations between treatment regimens and the risk of heart failure hospitalization, adjusting for potential confounders such as age, gender, LVEF, and hypertension. This analysis provided HRs for each treatment group intensified with SGLT2 inhibitors.

Ethical considerations

The protocol coded CEI-000001 by the Imbanaco Clinic was reviewed and approved by code CEI-953 on September 30, 2024. This study was classified as a no-risk investigation under Resolution 008430 of 1993 of the Colombian Ministry of Health, Resolution No. 2378 of 2008 of the Ministry of Social Protection of Colombia, the Guide to Good Clinical Practices (ICH-GCP E6), as well as its adherence to the principles of the World Medical Assembly outlined in the Declaration of Helsinki of 1964.

## Results

Population description

The study included 122 patients diagnosed with uncontrolled T2DM, categorized into five pharmacological treatment groups. The first group, consisting of 25 patients (20.5%), received a combination of metformin, glibenclamide, and dapagliflozin. The second group, comprising 25 patients (20.5%), was treated with metformin, saxagliptin, and dapagliflozin. The third group included 23 patients (18.9%) who received metformin along with exenatide and dapagliflozin, while the fourth group, consisting of 29 patients (23.8%), was treated with metformin, insulin, and dapagliflozin. Finally, the fifth group included 20 patients (16.4%) treated with metformin and empagliflozin.

The average age of the population was 55.9 years (SD ±7.6), with a prevalence of hypertension of 65.6% and an LVEF below 50% in 27% of the patients. Baseline values for HbA1c, weight, and blood pressure were similar across groups, with an average baseline HbA1c of 8.05% (SD ± 0.55), an average weight of 82.80 kg (SD ± 9.49), and an average SBP of 145.57 mmHg (SD ± 19.54) (Tables 1-2).

**Table 1:** Population description HbA1c: glycated hemoglobin, LVEF: left ventricular ejection fraction

Variable	n	%
Female	66	54.1
Male	56	45.9
Black	72	59.0
Mestizo	50	41.0
Hypertension	80	65.6
Baseline HbA1c ≥7%	122	100
HbA1c <7% at 18 months	62	50.8
LVEF <50%	33	27.0
Heart failure hospitalization	12	9.8

**Table 2:** Baseline characteristics HbA1c: glycated hemoglobin, SBP: systolic blood pressure, DBP: diastolic blood pressure, LVEF: left ventricular ejection fraction

Treatment group	n	Age (years)	Duration of diabetes (years)	Baseline HbA1c (%)	Baseline weight (kg)	Baseline SBP (mmHg)	Baseline DBP (mmHg)	Baseline LVEF (%)
Metformin + glibenclamide + dapagliflozin	25	55.56 ± 6.82	6.20 ± 2.74	8.04 ± 0.56	83.24 ± 9.29	150.68 ± 23.26	86.80 ± 7.27	52.28 ± 8.89
Metformin + saxagliptin + dapagliflozin	25	54.64 ± 8.77	7.36 ± 2.91	8.07 ± 0.54	80.88 ± 9.14	153.08 ± 21.30	90.04 ± 6.65	52.32 ± 6.30
Metformin + exenatide + dapagliflozin	23	53.78 ± 6.68	3.87 ± 2.36	8.03 ± 0.49	85.57 ± 8.62	144.74 ± 17.44	85.26 ± 8.23	52.17 ± 6.76
Metformin + insulin + dapagliflozin	29	56.90 ± 6.14	6.72 ± 3.28	8.25 ± 0.54	85.55 ± 9.86	145.45 ± 14.86	85.62 ± 7.08	51.59 ± 4.70
Metformin + empagliflozin	20	59.15 ± 9.05	3.75 ± 2.17	7.75 ± 0.56	77.50 ± 8.63	130.95 ± 12.74	83.70 ± 5.53	54.30 ± 5.47

HbA1c control and weight reduction by pharmacological group

**Figure 1:**
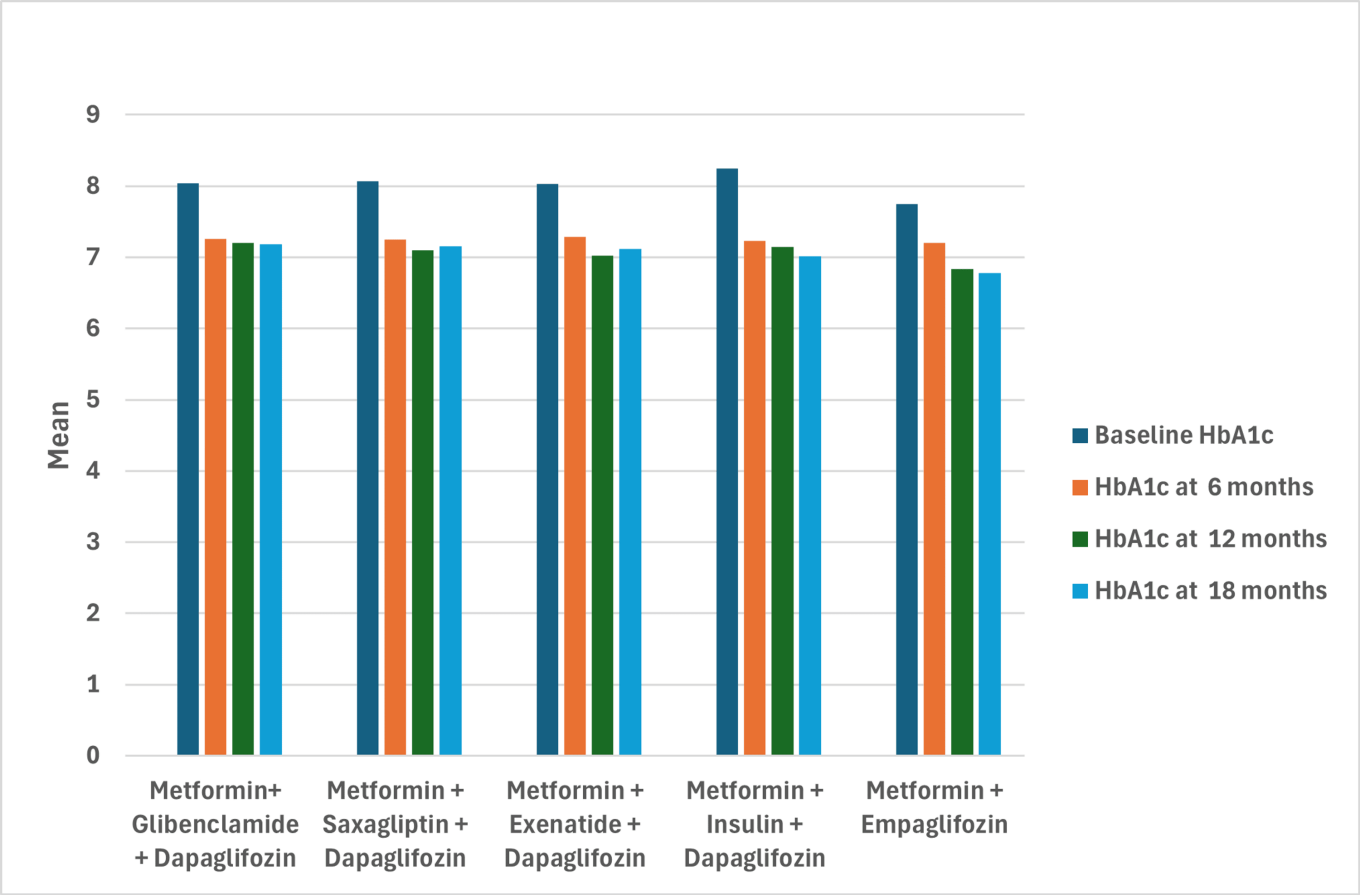
Mean HbA1c levels over time by treatment group HbA1c: glycated hemoglobin

Over the 18-month follow-up period, all treatment groups showed a significant reduction in HbA1c levels, with the most notable decrease observed in the empagliflozin group. HbA1c levels in the metformin + empagliflozin group decreased from an initial mean of 7.75% to 6.77% by the end of the study, with a reduction of -0.97% (95% CI: -1.31 to -0.63, p < 0.001) compared to baseline. In the other groups, final HbA1c values were 7.18% for metformin + glibenclamide + dapagliflozin, 7.16% for metformin + saxagliptin + dapagliflozin, 7.12% for metformin + exenatide + dapagliflozin, and 7.01% for metformin + insulin + dapagliflozin. A repeated-measures ANOVA demonstrated a significant reduction in HbA1c levels across all groups over time (F = 269.96, p < 0.001), with the empagliflozin group being the only one achieving HbA1c levels below 7% at the end of the follow-up (Figure 1).

**Figure 2:**
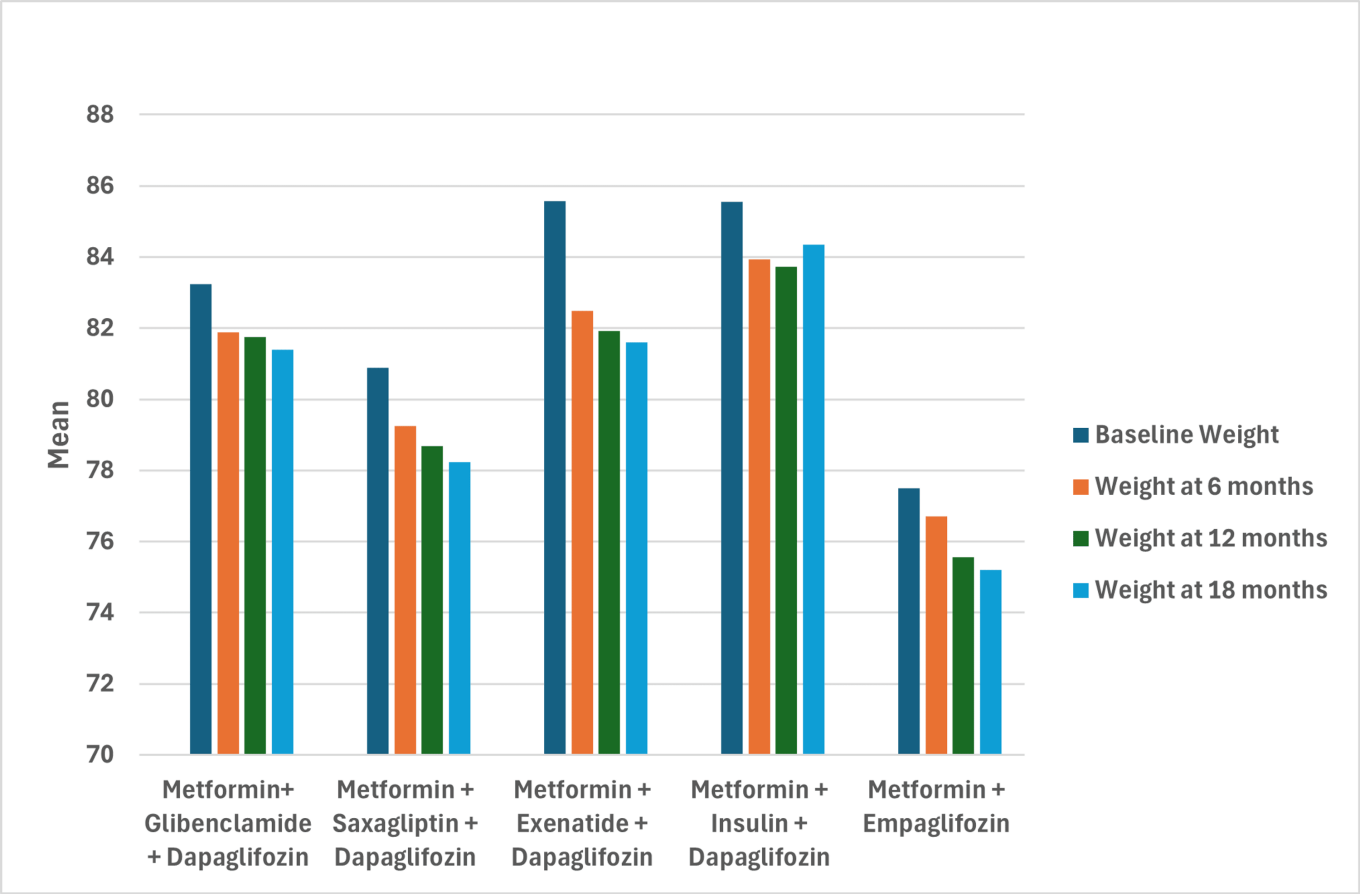
Mean weight changes over time by treatment group

Significant changes in weight reduction were also observed across all groups. In the metformin + empagliflozin group, mean weight decreased from 77.50 kg to 75.20 kg, corresponding to a reduction of -2.3 kg (p < 0.001). Other groups also experienced weight reductions: from 83.24 kg to 81.40 kg for metformin + glibenclamide + dapagliflozin, 80.88 kg to 78.24 kg for metformin + saxagliptin + dapagliflozin, 85.57 kg to 81.61 kg for metformin + exenatide + dapagliflozin, and 85.55 kg to 84.34 kg for metformin + insulin + dapagliflozin (p < 0.001 for all groups). The group with the greatest weight reduction was metformin + exenatide + dapagliflozin (-3.9 kg; 95% CI: -5.30 to -2.61, p < 0.001), followed by metformin + saxagliptin + dapagliflozin (-2.64 kg; 95% CI: -3.93 to -1.35, p < 0.001), both showing significant reductions over time compared to baseline (Figure 2).

Blood pressure control

**Figure 3:**
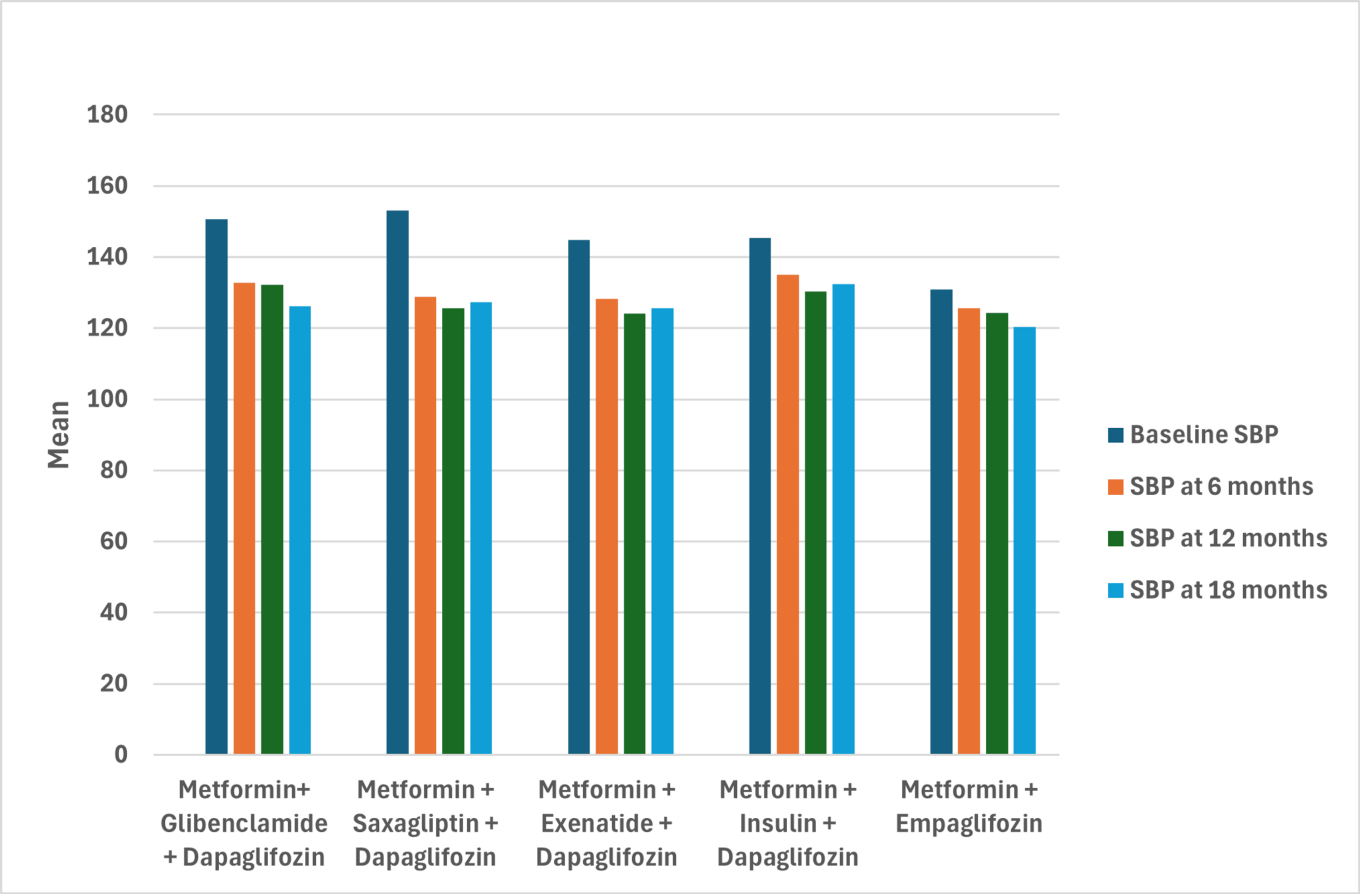
Mean SBP over time by treatment group SBP: systolic blood pressure

**Figure 4:**
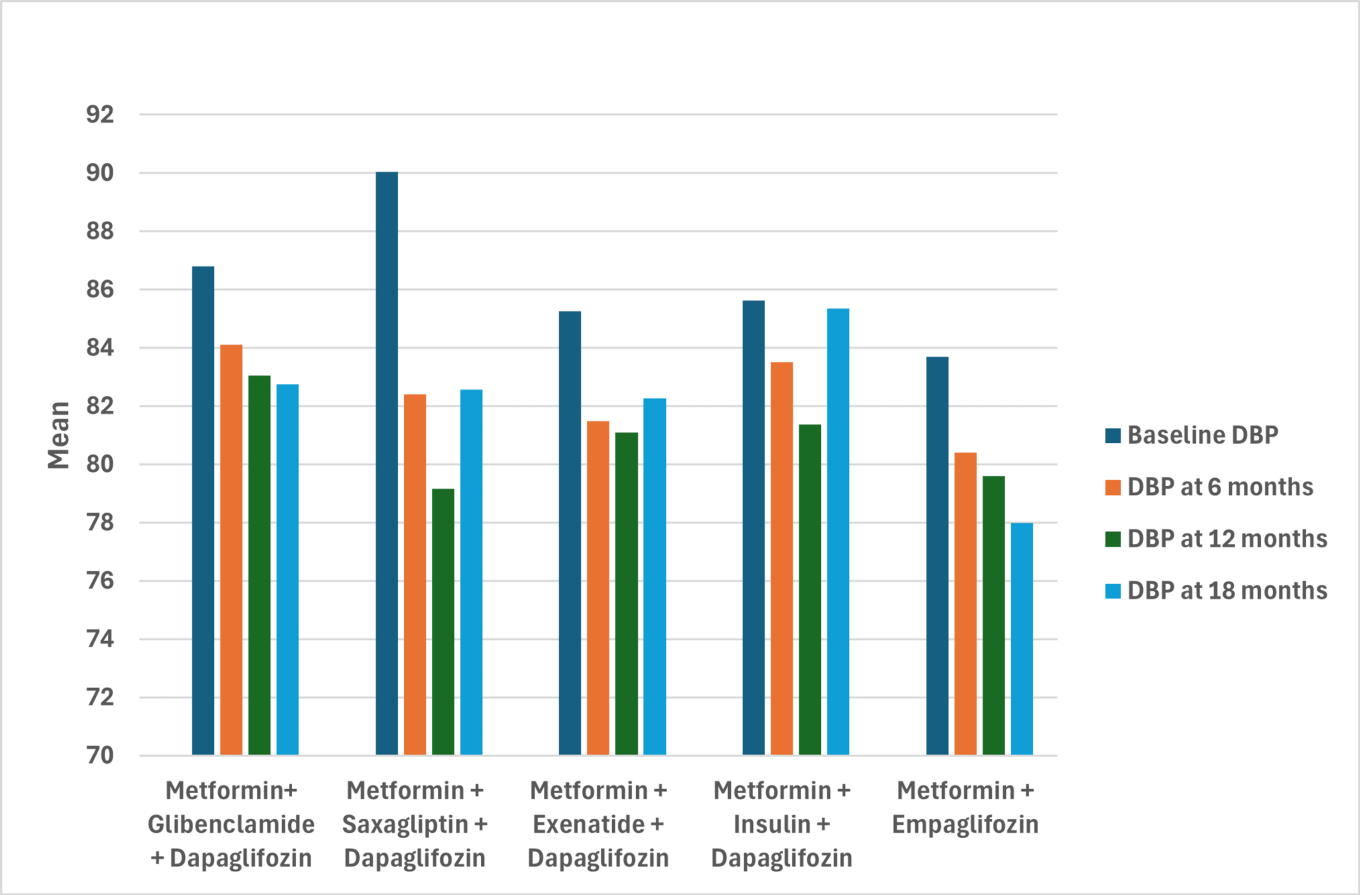
Mean DBP over time by treatment group DBP: diastolic blood pressure

Reductions in both SBP and DBP were observed across all groups, with the empagliflozin group showing the best blood pressure control. The final SBP in this group was 120.40 ± 6.11 mmHg after 18 months, representing a decrease from 130.95 mmHg to 120.40 mmHg. DBP also decreased from 83.70 mmHg to 78.00 mmHg. Although the greatest decrease in SBP was observed in the metformin + saxagliptin + dapagliflozin group (-25.84 mmHg; 95% CI: -36.65 to -15.03, p < 0.001), only the metformin + empagliflozin group achieved a final DBP <80 mmHg, with a decrease of -5.7 mmHg (95% CI: -10.71 to -0.69, p < 0.05) (Figures 3-4).

Heart failure hospitalization

During the follow-up period, 12 hospitalizations due to heart failure were recorded, representing an overall hospitalization rate of 9.8%. In the metformin + empagliflozin group, no hospitalizations (0%) were reported. In contrast, the groups treated with combinations of dapagliflozin showed hospitalization rates of 16% (metformin + glibenclamide + dapagliflozin), 12% (metformin + saxagliptin + dapagliflozin), 13% (metformin + exenatide + dapagliflozin), and 7% (metformin + insulin + dapagliflozin).

**Figure 5:**
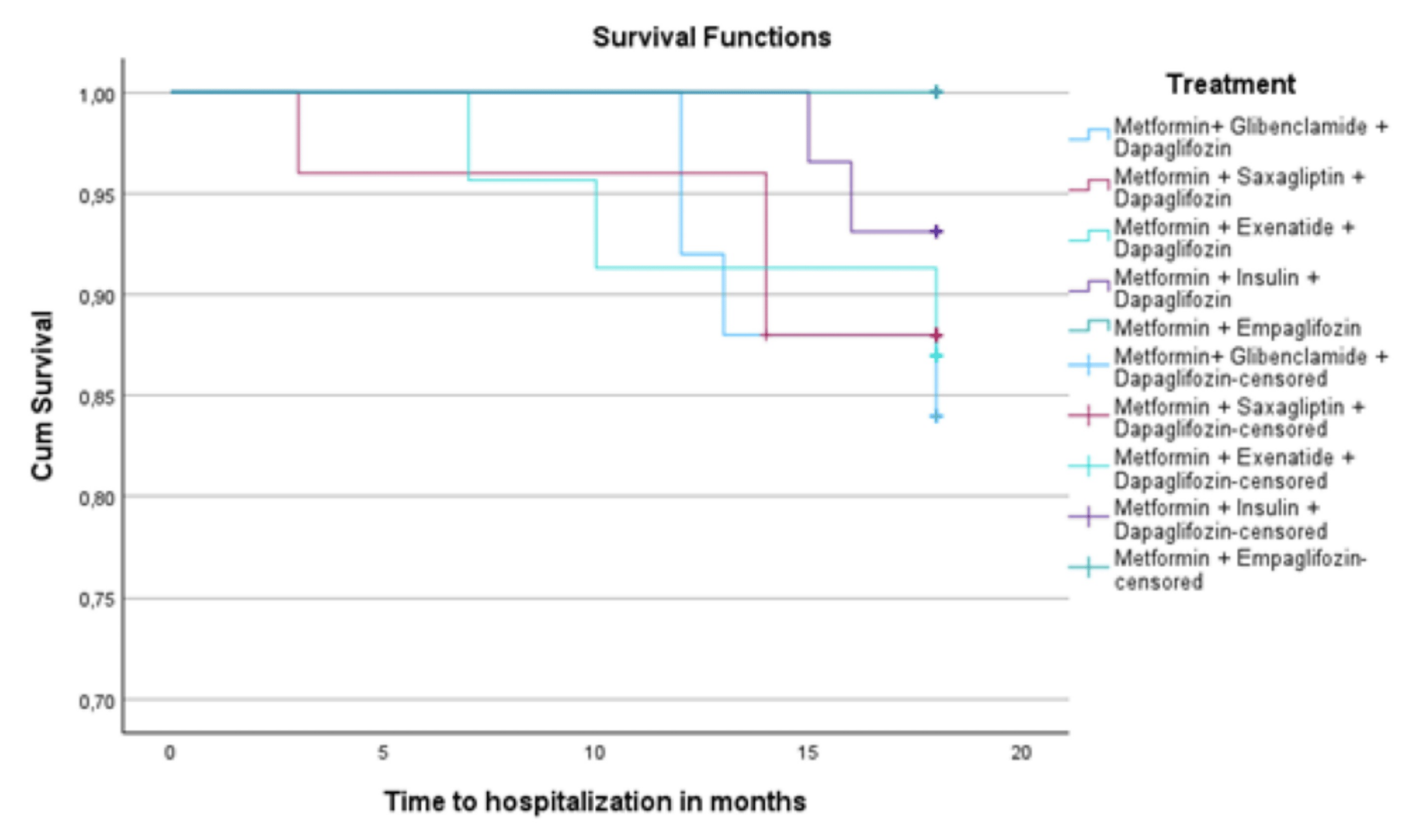
Kaplan-Meier survival curve for time to heart failure hospitalization by treatment group

**Figure 6:**
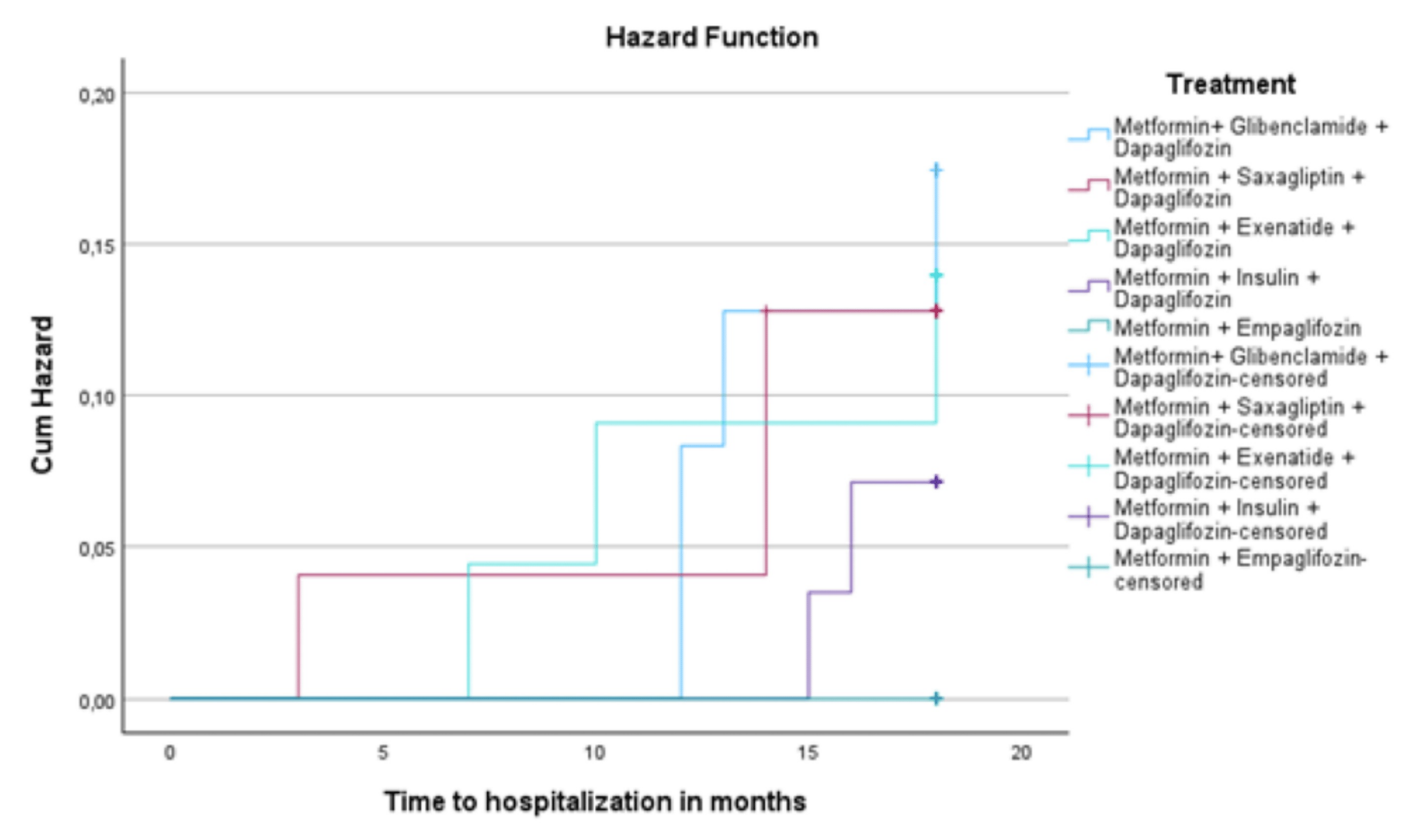
Cumulative hazard function for time to heart failure hospitalization by treatment group

**Figure 7:**
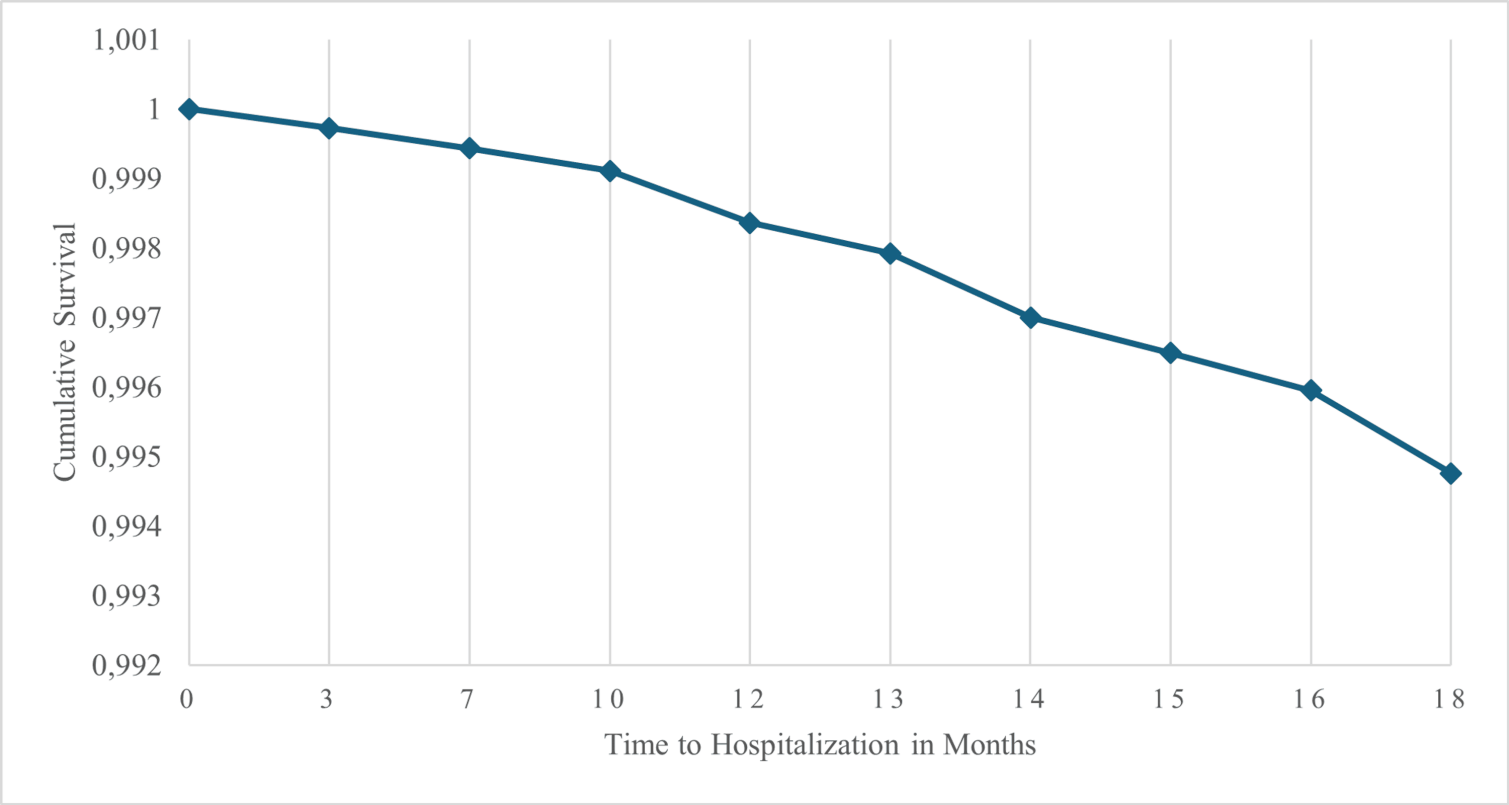
Cumulative survival curve for time to hospitalization in months

Kaplan-Meier survival analysis indicated a trend toward greater hospitalization-free survival in the empagliflozin group. However, the difference did not reach statistical significance in the log-rank test (Mantel-Cox, p = 0.064). Cox regression analysis revealed a preventive impact of empagliflozin treatment (Figures 5-7). For the empagliflozin group, the HR was 0 (p = 0.98), corresponding to the absence of heart failure hospitalizations throughout the study period.

Cox regression also adjusted for other variables, including age, gender, LVEF, and hypertension. Patients with an LVEF <50% had an increased risk of hospitalization (HR = 4.53; 95% CI: 1.07-19.21; p = 0.040). DBP at 18 months was also significant in the model (HR = 1.15; 95% CI: 1.01-1.32; p = 0.039), indicating that a 1 mmHg increase in DBP was associated with a 15.1% increase in the risk of hospitalization.

HbA1c levels at 18 months were not statistically significant (HR = 3.30; 95% CI: 0.55-19.91; p = 0.193), although higher levels were suggested to be associated with an increased risk. This effect was inconclusive in this analysis.

The estimated survival function from the model showed a progressive decline in the probability of remaining hospitalization-free over the 18 months of follow-up (Figure 7). By 18 months, the cumulative survival probability was 90.2%, indicating that 9.8% of patients experienced hospitalizations during this period.

**Figure 8:**
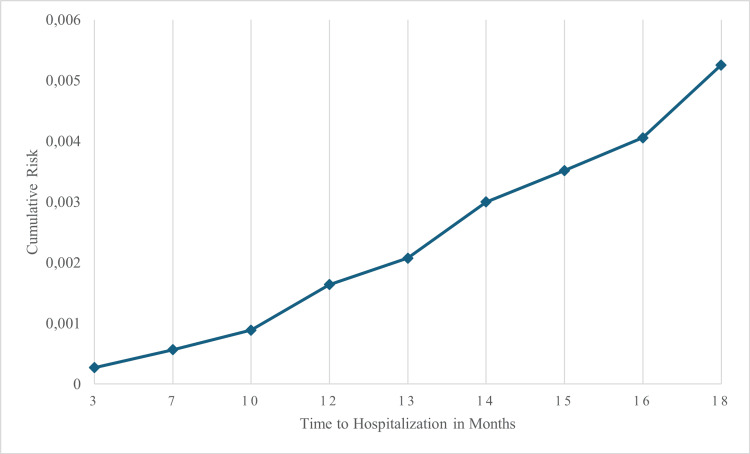
Cumulative risk curve for heart failure hospitalization by treatment group

In contrast, the cumulative risk function (Figure 8) showed a gradual increase in risk over time, becoming more pronounced after 12 months. This suggests that the risk of hospitalization increases with time, particularly toward the end of the study period.

## Discussion

In patients with T2DM, the lifetime risk of developing cardiovascular disease by the age of 50 is 67% for men and 57% for women, with macroangiopathy observed in up to 40% of cases at diagnosis. In this study, the patients averaged 50 years of age, with disease durations consistent with elevated cardiovascular risk despite not being classified as such or having been diagnosed with heart failure. All patients underwent intensified pharmacological management with SGLT2 inhibitors, predominantly dapagliflozin and empagliflozin, while maintaining their pre-existing diabetes medications [21].

Glycemic control and metabolic benefits

All patient groups had HbA1c levels above 7% when initiating SGLT2 inhibitors. From the outset, sustained reductions in HbA1c were observed across all groups undergoing treatment intensification, with significant decreases in all groups. Empagliflozin demonstrated the most notable improvement, reducing HbA1c from a baseline of 7.75% to 6.77% at the end of 18 months (95% CI: 0.8-2.0; p < 0.001), becoming the only group to achieve HbA1c levels below 7% at follow-up. These results align with findings from the EMPA-REG study, where empagliflozin achieved reductions of approximately 0.5% in high-risk patients, with amplified effects when combined with metformin, as reported reductions of up to 2.05% after three months of therapy [6,9,22].

Weight reduction

Sustained weight loss was observed across all treatment groups using SGLT2 inhibitors, with statistically significant reductions from baseline in all groups. The greatest weight reduction occurred in the metformin + exenatide + dapagliflozin group (-3.9 kg; 95% CI: -5.30 to -2.61, p < 0.001). In the empagliflozin group, the average weight reduction was -2.3 kg over 18 months (95% CI: -3.74 to -0.86, p < 0.001), resulting in the lowest final weight among the groups (75.20 ± 8.54 kg). This reduction is particularly significant for glycemic control and mitigating the burden of obesity in patients who do not necessarily present high cardiovascular risk, helping to prevent long-term metabolic and cardiovascular complications.

These findings are consistent with a meta-analysis of 34 randomized clinical trials involving 9,154 patients, comparing SGLT2 inhibitors to placebo. The analysis reported an average weight reduction of 2 kg (1.7-2.9 kg) over 12 weeks. This supports the early use of SGLT2 inhibitors as part of an integral approach to T2DM management, particularly in overweight or obese patients at higher risk of disease progression, emphasizing the role of empagliflozin in patients without documented high cardiovascular risk [23,26].

Blood pressure control

All groups treated with SGLT2 inhibitors showed reductions in SBP and DBP over time. The most significant reduction for DBP was observed in the metformin + saxagliptin + dapagliflozin group, followed by the metformin + empagliflozin group. At 18 months, the metformin + empagliflozin group achieved the best control, with a DBP of 78 ± 4.19 mmHg, the only group with DBP <80 mmHg.

Regarding SBP, the largest reduction was observed in the metformin + saxagliptin + dapagliflozin group. In the metformin + empagliflozin group, the reduction was -10.55 mmHg (95% CI: -22.64 to -1.53, p < 0.13), with a final SBP of 120.40 ± 6.11 mmHg at 18 months, representing the best blood pressure control at the end of the follow-up. These findings suggest that empagliflozin effectively improves hemodynamics in diabetic patients without established cardiovascular disease. These results are consistent with but exceed those reported in the literature, which documents reductions of 4 mmHg for SBP and 2 mmHg for DBP [23,25,27].

LVEF below 50% was a significant predictor of hospitalization (HR = 4.53; 95% CI: 1.07-19.21, p = 0.040). This result is consistent with findings from EMPEROR-Reduced, where empagliflozin demonstrated a significant benefit in reducing hospitalizations among patients with reduced ejection fraction, regardless of the presence of diabetes. Prior studies suggest that the therapeutic benefits of medications for heart failure, such as SGLT2 inhibitors, tend to become more evident as ventricular function deteriorates. This highlights the potential for empagliflozin to provide pronounced protective effects in patients with left ventricular dysfunction [23,28].

Prevention of long-term complications and heart failure hospitalization - reduction in heart failure hospitalizations

Studies such as EMPEROR-Preserved, EMPEROR-Reduced, and DELIVER have reported heart failure hospitalization rates of 13.8%, 19.4%, and 16.4%, respectively, in patients treated with SGLT2 inhibitors compared to placebo. This study's event rate was 9.8%, primarily in patients with preserved ejection fraction, with no deaths recorded.

Regarding heart failure hospitalizations, empagliflozin demonstrated an advantage with an HR of 0 (p = 0.98), although without statistical significance. In previous studies, empagliflozin has also shown a risk reduction in patients with prior heart failure. However, our findings highlight its preventive potential in early-stage T2DM patients with no cardiovascular disease history, suggesting that empagliflozin could be integrated into standard treatment to reduce the risk of developing heart failure over time [26,27].

In this analysis, sex and race were not statistically significant in the Cox regression model (p > 0.05). For sex, the HR was 2.38 (95% CI: 0.58-9.75, p = 0.23), indicating a potential, but inconclusive, increased risk of hospitalization in men. This finding aligns with EMPA-REG OUTCOME, which also reported reductions in cardiovascular events with empagliflozin regardless of sex or race [9,22].

Glycemic control and heart failure risk

HbA1c levels at 18 months showed an HR of 3.30 (95% CI: 0.55-19.91, p = 0.193), suggesting a possible association between elevated HbA1c levels and hospitalization risk. However, this effect was not statistically significant in our study. Similar findings have been reported in EMPEROR-Reduced, where glycemic control was relevant but not the primary driver of risk reduction in patients treated with empagliflozin. This suggests that the protective effects of empagliflozin may be mediated by mechanisms beyond glycemic control [23,28].

Impact of blood pressure on risk

DBP at 18 months was a significant variable in the model (HR = 1.15; 95% CI: 1.01-1.32, p = 0.039), indicating that a 1 mmHg increase in DBP was associated with a 15.1% increase in the risk of heart failure hospitalization. In contrast, SBP was not statistically significant (HR = 1.03, p = 0.553). These findings are noteworthy as other studies, such as EMPEROR-reserved, have also found that the benefits of empagliflozin in heart failure are not exclusively dependent on blood pressure changes. However, DBP control may play a role in preventing adverse events [23,27].

Diabetes duration and hospitalization risk

The duration of diabetes was significant in the model (HR = 0.71; 95% CI: 0.53-0.95, p = 0.021). This finding suggests that longer disease duration is associated with a lower hospitalization risk, potentially reflecting better adaptation to intensified treatments or greater awareness and control of risk factors over time. Although the literature on the impact of disease duration on heart failure hospitalization risk is limited, studies such as EMPA-REG OUTCOME underscore that early empagliflozin initiation could be an effective strategy to reduce adverse events in T2DM, reinforcing the need for early intervention [22].

Comparison with classical studies

Our findings underscore the effectiveness of empagliflozin, which is consistent with large-scale studies such as EMPA-REG and CANVAS in reducing cardiovascular events. However, a key difference lies in the focus of these studies on patients with high cardiovascular risk. In contrast, the benefits observed in this study suggest that empagliflozin can also be valuable in the early stages of T2DM before severe cardiovascular comorbidities develop. The HRs in our cohort, with no high-risk comorbidities, were similar to those in previous studies (HR of 0.65 in EMPA-REG for heart failure hospitalization), highlighting that the protective effects of empagliflozin could broadly benefit the general T2DM population [6,22].

Ejection fraction as a predictor of cardiovascular events

LVEF was used as an important marker in this study, with a threshold value of <50% to classify patients at higher risk for cardiovascular events. Our analysis found that an LVEF <50% was associated with an increased risk of heart failure hospitalization, with an HR of 4.53 (95% CI: 1.07-19.21, p = 0.040), a result that reached statistical significance. This association was also observed in large studies such as EMPEROR-Reduced, DAPA-HF, and DELIVER, which suggest that patients with reduced LVEF especially benefit from treatments such as empagliflozin and dapagliflozin, which in these studies demonstrated a protective effect on cardiac function and reduced hospitalization rates in patients with reduced heart failure. Notably, in our study, none of the patients with reduced LVEF in the empagliflozin group experienced cardiovascular events during follow-up. Additionally, regardless of LVEF, no events of heart failure hospitalization or deaths were recorded in the empagliflozin group. However, it is important to note that the total number of patients receiving empagliflozin was smaller than in other groups, and their baseline HbA1c levels were slightly lower at the start of the study. These factors may have contributed to the observed outcomes and should be considered when interpreting the findings [26,28,29].

First heart failure hospitalization and the effect of empagliflozin

Regarding the first heart failure hospitalization, patients treated with empagliflozin showed a lower incidence of events than other therapeutic groups. The heart failure hospitalization rate in the empagliflozin group was 0%, while the hospitalization rate in the dapagliflozin combined with other agents group ranged between 7% and 16%. The Kaplan-Meier analysis and log-rank test showed a trend toward a lower probability of events in the empagliflozin group. However, no statistically significant difference was observed in this analysis (p = 0.064). However, this trend is clinically relevant and agrees with studies like EMPA-REG, which reported a 35% relative risk reduction in heart failure hospitalization in high cardiovascular risk patients and 20% in patients with preserved ejection fraction. The key distinction in our study is that no events were reported in the metformin + empagliflozin group, regardless of hypertension history or even in patients with reduced LVEF. Nevertheless, it is important to emphasize that the total number of patients included in the empagliflozin group was smaller than those receiving dapagliflozin. This difference in group size may have influenced the results and should be considered when comparing outcomes between treatment groups [22,26].

Comparison of HRs with classical studies

The use of SGLT2 inhibitors has been documented in studies such as DAPA-HF and EMPEROR-Reduced, where significant reductions in heart failure hospitalization risk (HR = 0.75 and HR = 0.70, respectively) were reported in patients with prior heart failure, as well as in patients with preserved or moderately reduced ejection fraction. Similarly, studies like DELIVER (HR = 0.82) and EMPEROR-preserved (HR = 0.79) have documented reduced heart failure hospitalizations. Our findings suggest that empagliflozin may offer protective benefits even in those without a history of heart failure or severe comorbidities. The results indicate an HR of 0, p > 0.05, reflecting no death or hospitalization for heart failure occurrences in this group, although limited by sample size. This suggests a similar or even greater reduction in hospitalization risk in patients at the early stages of the disease, where, despite an average age of 50 years, there is already elevated cardiovascular risk, reinforcing the idea that empagliflozin can be applied as an effective preventive treatment, alongside metformin, from the onset of the disease in adult patients, regardless of whether they meet the latest ADA guidelines, which restrict treatment to those with documented high cardiovascular risk. This is especially important since prediabetes already shows elevated risks compared to the general population, and 40% of patients have some form of macrovascular complication at diagnosis [7,30].

Implications of early empagliflozin use in patients without documented high cardiovascular risk

Ejection fraction and first heart failure hospitalization have proven to be key indicators for identifying patients at risk of heart failure progression. However, the high documented cardiovascular risk does not exclude the existence of pre-existing cardiovascular risk from prediabetic stages, as evidenced in studies like CHARM, ICFER, and PARADIGM-HF, in which 22-25% of patients with reduced ejection fraction heart failure were prediabetic, and 13-26% were undiagnosed T2DM patients. The absence of hospitalization events in the empagliflozin-treated group in this sample suggests that early use of this medication could prevent heart failure progression in a broader group of patients beyond those with high cardiovascular risk or existing heart dysfunction. This suggests empagliflozin not only as a treatment option for at-risk patients but also as an early intervention that could potentially modify the natural progression of diabetes and its cardiovascular complications, focusing on prevention from the first years after diagnosis [21,30,31].

Strengths and limitations

The real-world follow-up between treatment groups is a valuable strength of these findings. However, his study has inherent limitations. Its retrospective design may introduce selection bias and the influence of unmeasured confounders despite efforts to adjust for key variables. Additionally, the relatively small sample size and low number of events limit the statistical power and generalizability of the findings. Future research with larger population groups, prospective designs, and complementary statistical approaches is recommended to validate and expand upon the results observed in this analysis. These efforts are crucial to strengthen the evidence and provide a broader understanding of the impact of SGLT2 inhibitors in diverse clinical settings.

## Conclusions

The analysis of LVEF and first heart failure hospitalization in patients with T2DM treated with SGLT2 inhibitors reveals that empagliflozin is not only effective in improving glycemic control, weight, and blood pressure, but also demonstrates preventive potential in heart failure progression, even in patients without documented high cardiovascular risk.

These findings, aligned with evidence from classical studies, suggest that empagliflozin could be considered in the early management of T2DM to reduce the incidence of heart failure and improve long-term outcomes. Furthermore, this study proposes recognizing patients with T2DM as high-risk from the early stages of the disease, making them candidates for combined therapy with SGLT2 inhibitors.
